# Optic cupping secondary to periventricular leukomalacia: A potential mimic for normal pressure glaucoma

**DOI:** 10.1016/j.radcr.2022.07.106

**Published:** 2022-09-11

**Authors:** Ahmed H El Beltagi, Nour Barakat, Loai Aker, Laith Abandeh, Ahmed Own, Mohamed Abdelhady, Hassan Aboughalia

**Affiliations:** a Neuroradiology Section, Neuroscience Institute, Hamad Medical Corporation, Weill Cornell Medicine, Doha, Qatar; b Ophthalmology Department, Hamad Medical Corporation, Doha, Qatar; c Department of Clinical Imaging, Hamad Medical Corporation, P.O box 3050, Doha, Qatar; d Department of Radiology, University of Washington, Seattle, WA, USA; e Neuroradiology Section, Neuroscience Institute, Hamad Medical Corporation, Doha, Qatar; f Radiology Department, Seattle Children's Hospital, University of Washington Medical Center, Seattle, WA, USA

**Keywords:** Periventricular leukomalacia, Optic disk, Normal pressure glaucoma

## Abstract

We herein present a case of periventricular leukomalacia (PVL) with secondary optic pathway denervation atrophy, which was initially labeled as normal tension glaucoma. However, given the discordant clinical and ophthalmologic findings, brain magnetic resonance imaging was requested which proved PVL to be the underlying process to the patient's decreased visual acuity. In addition to presenting the ophthalmologic findings, we are emphasizing the pivotal role of neuroimaging in ruling out central causes of optic atrophy/hypoplasia and making this clinical distinction by demonstrating optic pathway atrophy associated with PVL.

## Introduction

In patients with decreased visual acuity, ophthalmologic evaluation is the initial assessment step. The appearance of the optic disk, the exit point for ganglion cell axons leaving the eye, is essential in such evaluation. In patients with decreased visual acuity and associated abnormal cup to disk ratio, glaucoma is the initial diagnostic consideration. However, imaging can play a central role excluding central brain disorders mimicking glaucoma. Radiologists shall be aware of the potential brain pathologies that clinically present in a glaucoma-like fashion. On the other hand, it is imperative for the ophthalmologists to recognize the clues that suggest an alternative diagnosis to avoid the burden of inappropriate management.

## Case report

A 21-year-old gentleman presented to the ophthalmology clinic with decreased visual acuity, more severe on the right side. Five years back, he was diagnosed with normal tension glaucoma and was prescribed topical prostaglandin analogue and combination topical carbonic anhydrase inhibitors-beta blockers. Despite compliant treatment, his conditioned deteriorated. This prompted a new referral to our clinical for further ophthalmologic evaluation.

**Fig. 1:**
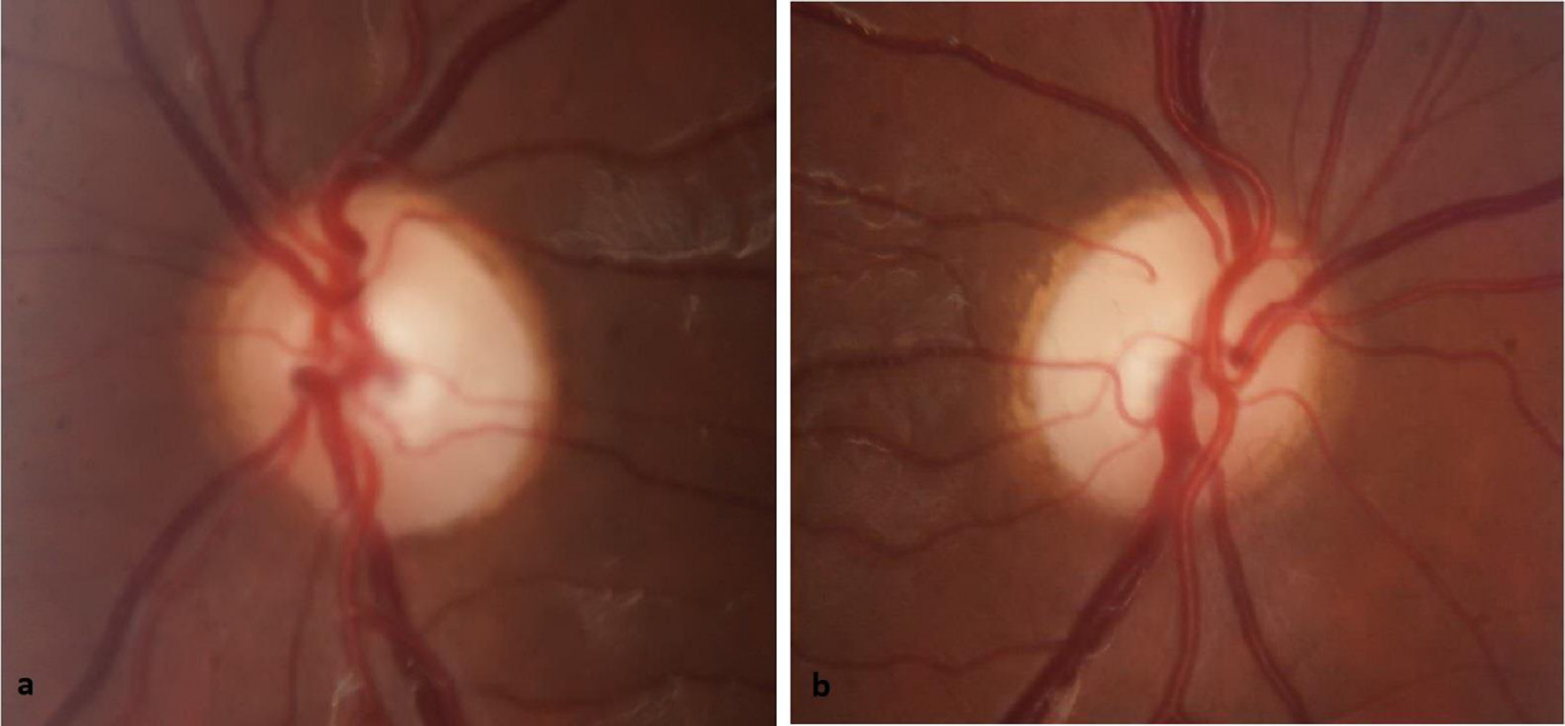
Visual field perimetry 24-2, showing typical bilateral inferior visual field defect, a classical finding in patients with PVL.

**Fig. 2:**
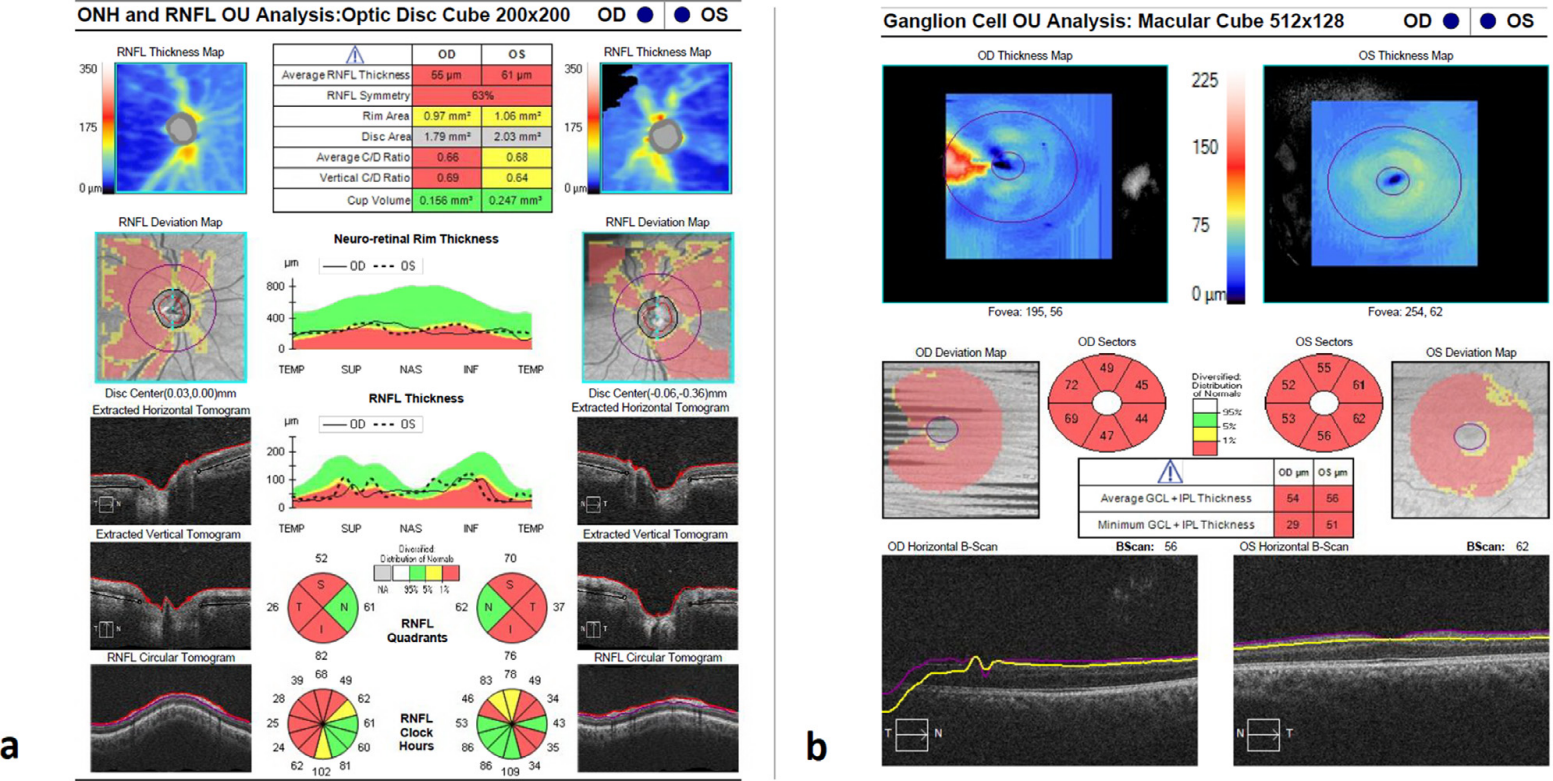
(A) Optical coherence tomography image showing severe bilateral retinal nerve fiber layer (RNFL) loss in both eyes, (B) profound ganglion cell layer loss (GCC) with healthy appearing outer retinal layers suggesting a primary neuronal loss rather than a secondary loss due to retinal disease.

Our patient's past ophthalmologic history was remarkable for childhood squint surgery as well as bilateral visual impairment. Co-existing medical conditions included diabetes mellitus, hypertension, and dyslipidemia. Initial ophthalmologic examination revealed that the best corrected visual acuity was 6/36 for the right eye and 6/12 for the left eye. The intraocular pressure was 14 mmHg and gonioscopic exam was normal. The eye fundus was evaluated following appropriate pupillary dilatation and showed rounded normal size optic disk. However, the cup was deep and large bilaterally yielding a cup to disk (C/D) ratio of 0.5 on the right side and 0.7 on the left side. In addition, there was significant circumferential thinning in the neuroretinal rim (Fig. 1). Further evaluation using optical coherence tomography was performed and showed significant loss in the retinal nerve fiber layer (Fig. 2).

**Fig. 3:**
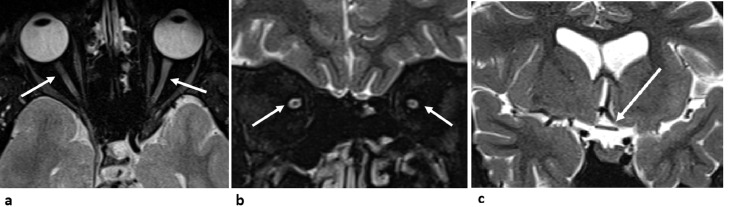
(A) Axial and (B) coronal T2WI fat-saturated images showing bilateral significant attenuation/atrophy of optic nerve/sheath complex (arrows in A, and B). (C) Coronal T2WI fat-saturated images showing attenuated/atrophic optic chiasm (arrow).

**Fig. 4:**
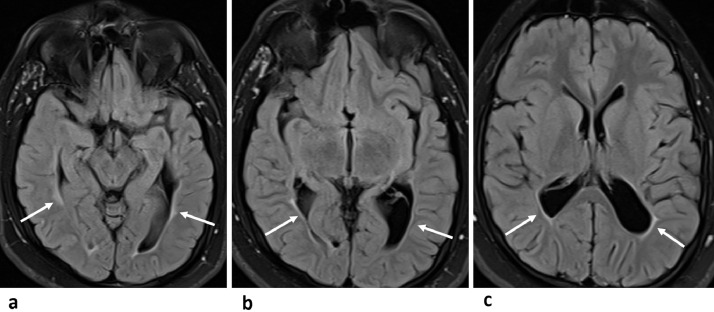
(A-C) Axial FLAIR-T2W images from inferior to showing evidence of posterior predominant volume loss of deep periventricular white matter, with ex-vacuo dilatation of the atria-trigones, temporal, and occipital horns of the lateral ventricles on both sides with irregular wavy outline, and peri-ventricular bright signal intensity gliotic changes abutting the ependymal surface (arrows in A-C).

The aggregate findings of this comprehensive ophthalmologic evaluation again suggested a diagnosis of normal tension glaucoma. However, the patient age as well as the disease progression despite adequate treatment raised the possibility of an alternative central pathology. Hence, nonenhanced magnetic resonance imaging (MRI) of the head and orbits was pursued. Orbital evaluation on the MRI showed diffuse thinning of optic nerve sheath bilaterally extending to the optic chiasm (Fig. 3). Also, the lateral ventricles had wavy contours with ex-vacuo dilatation of their atria, trigones, temporal, and occipital horns secondary to white matter volume loss, most prominent within the occipital lobes. There were gliotic changes exhibiting the classic periventricular T2WI high signal intensity (Fig. 4). MRI features were suggestive of perinatal ischemic insult with resultant periventricular leukomalacia (PVL) involving the optic radiation. This would account for the patient's symptoms and the findings on the ophthalmologic evaluation; accordingly, the patient was diagnosed with nonglaucomatous visual field loss related to PVL. The patient was advised to stop antiglaucoma medications, and further follow-up for almost 2 years showed no progression of visual field defect.

## Discussion

PVL is a known phenomenon in the context of perinatal insult in premature babies [1,2]. It is postulated that PVL develops during the third trimester of pregnancy mainly following hypoxic ischemic injury but can also result from infective or inflammatory processes [1,[3], [4], [5].

PVL preferentially involves the deep periventricular white matter tracts in the actively myelinating tracts in the peritrigonal area. This results in the characteristic ex-vacuo dilatation of the ventricles with wavy ventricular outline [2,^5^,6]. The optic radiation is responsible for relaying the visual information from the lateral geniculate bodies to the visual cortex [3,^5^,7]. When PVL involves the optic radiation, the axonal disruption results in trans-synaptic denervation and atrophy. Depending on the time of injury, optic radiation axonal interruption results in 2 distinct patterns of optic nerve injury in premature infants. Injury at an earlier age results in optic nerve axonal loss, with small optic disk area, and a small cup, because the plasticity of the sclera at this stage of development allows for remodeling of the optic nerve shape after axonal loss. This is in contrast to injury following the establishment of scleral canals secondary to trans-synaptic degeneration of the retinogeniculate axons which results in reduced neuroretinal rim and large cups with normal disk, that appears similar to that seen in normal pressure glaucoma [3,5].

Although PVL is not a rare finding in neuroimaging, the added value of linking the patient clinical data to their imaging findings is substantial. Findings on MRI that might suggest an alternative etiology to patient's clinical diagnosis of glaucoma include PVL, predominantly involving the optic radiation, and optic pathway atrophy [4]. Appropriate communication of these findings to the referring ophthalmologist helps in clenching the proper diagnosis and adjusting their patients’ management plan.

On the other hand, it is essential to the treating ophthalmologist to carefully evaluate patients’ medical history, including perinatal history. If such history is overlooked, the relationship of optic disk cupping, field defects, with possible prematurity can lead to a potential misdiagnosis with normal tension glaucoma. This is especially important as advances in perinatal care improved survival rates among premature infants, who may present later in life with visual field defects, nystagmus and strabismus.

Additional clues to a potential misdiagnosis of normal tension glaucoma include younger patient age as well as bilateral inferior defects respecting the horizontal midline on perimetry. This contrasts with the older age and the visual field defects corresponding to the appearance of the optic disk typically seen in patients with normal tension glaucoma [3,4]. These patients whose clinical picture or ophthalmologic evaluation is atypical can benefit from a more comprehensive workup including radiologic imaging to exclude central causes of visual impairment [8].

## Conclusion

It is essential to differentiate glaucoma from other pathologic entities that present with glaucoma-like (pseudo-glaucomatous) ophthalmologic picture. Our case illustrates how PVL can rarely induce optic disk changes that might mimic the more common entity of normal tension glaucoma. Although both conditions might have similar clinical presentation, they dictate a disparate management plan. Thus, awareness of this clinical scenario is crucial to both radiologists and ophthalmologists who are involved in the care of these patients.
